# Access criteria for anti-TNF agents in spondyloarthritis: influence on comparative 1-year cost-effectiveness estimates

**DOI:** 10.1186/s12962-017-0081-8

**Published:** 2017-09-07

**Authors:** Stephanie Harvard, Daphne Guh, Nick Bansback, Pascal Richette, Alain Saraux, Bruno Fautrel, Aslam Anis

**Affiliations:** 1 University of British Columbia, Vancouver, Canada; 2 Centre for Health Evaluation and Outcome Sciences, 588-1081, Burrard Street, Vancouver, BC V6Z 1Y6 Canada; 3 Sorbonne Universités, UPMC-GRC08, Pierre Louis Institute for Epidemiology and Public Health, Paris, France; 4 University Paris Diderot, Faculty of Medicine, Paris 07, Paris, France; 5 AP-HP, Rheumatology Department, Lariboisiere University Hospital, Paris, France; 6 Rheumatology Unit, Hôpital de la Cavale Blanche, 29609 Brest, France; 7 EA2216, INSERM ESPRI ERI29 Université de Brest LabEx IGO, Brest, France; 8 AP-HP, Rheumatology Department, Pitié Salpétrière University Hospital, Paris, France

**Keywords:** Cost-effectiveness, Anti-TNF, Biologics, Pharmaceutical policy, Spondyloarthritis

## Abstract

**Background:**

Anti-tumor necrosis factor (anti-TNF) agents are an effective, but costly, treatment for spondyloarthritis (SpA). Worldwide, multiple sets of access criteria aim to restrict anti-TNF therapy to patients with specific clinical characteristics, yet the influence of access criteria on anti-TNF cost-effectiveness is unknown. Our objective was to use data from the DESIR cohort, a prospective study of early SpA patients in France, to determine whether the French anti-TNF access criteria are the most cost-effective in that setting relative to other potential restrictions.

**Methods:**

We used data from the DESIR cohort to create five study populations of patients meeting anti-TNF access criteria from Canada, France, Germany, United Kingdom, and Hong Kong, respectively. For each study population, we calculated the costs and quality-adjusted life years (QALYs) over 1 year of patients treated and not treated with anti-TNF therapy. To control for differences between anti-TNF users and non-users, we used linear regression models to derive adjusted mean costs and QALYs. We calculated incremental cost-effectiveness ratios (ICERs) representing the incremental cost per additional QALY gained by treating with an anti-TNF within each of the five study populations, using bootstrapping to explore the range of uncertainty in costs and QALYs. A series of sensitivity analyses was conducted, including one to simulate the effect of a 24-week stopping rule for anti-TNF non-responders.

**Results:**

Anti-TNF access criteria from France were satisfied by the largest proportion of DESIR patients (27.8%), followed by Germany (25.1%), Canada (23.8%), the UK (12.1%) and Hong Kong (8.6%). Confidence intervals around incremental costs and QALYs in the basecase analysis were overlapping, indicating that anti-TNF cost-effectiveness estimates derived from each subset were similar. In the sensitivity analysis that examined the effect of excluding costs accumulated past 24 weeks by anti-TNF non-responders, the incremental cost per QALY was reduced by approximately 25% relative to the basecase analysis (France: €857,992 vs. €1,105,859; Canada: € 626,459 vs. €818,186; Germany: € 422,568 vs. €545,808); UK €578,899 vs. €766,217; Hong Kong €335,418 vs. €456,850).

**Conclusions:**

Anti-TNF cost-effectiveness is strongly affected by treatment continuation among non-responders. Access criteria could improve anti-TNF cost-effectiveness by defining patients likely to respond.

**Electronic supplementary material:**

The online version of this article (doi:10.1186/s12962-017-0081-8) contains supplementary material, which is available to authorized users.

## Background

Anti-tumour necrosis factor alpha (anti-TNF) agents, including infliximab, etanercept, adalimumab, golimumab, and certolizumab pegol, significantly reduce disease activity and improve functional ability among patients with spondyloarthritis (SpA), including ankylosing spondylitis (AS) and non-radiographic axial SpA (nr-axSpA) patients [1–3]. However, because of their similar high cost and potential side-effects, most health systems worldwide restrict access to all anti-TNF agents to SpA patients meeting specific clinical criteria. Van den Berg et al. described 23 different criteria sets from various international settings that designate which SpA patients are eligible to receive anti-TNF therapy [4]. Some of these criteria sets represent clinical recommendations, while others are reimbursement criteria. All of the criteria sets differ in terms of the diagnosis, disease activity level, and history of treatment failure required to begin anti-TNF therapy. Some criteria sets limit anti-TNF agents to patients with AS, a severe form of SpA in which bone damage is visible on X-ray; others approve anti-TNF use among patients with nr-axSpA, the term for SpA prior to the development of this radiographic damage. Many criteria sets that allow anti-TNF use by nr-axSpA patients require them to have sacroiilitis or spinal inflammation visible on MRI and/or elevated acute-phase reactants, such as C-reactive protein (CRP) or erythrocyte sedimentation rate (ESR), while others do not incorporate these additional markers.

The variation in these criteria sets means that patient access to anti-TNF therapy is more difficult in some settings than others. For example, only an estimated 50% or less of all SpA patients have AS [5], meaning far fewer SpA patients will be treated with anti-TNF therapy in settings that require radiographic damage. The prevalence of other clinical criteria commonly cited in anti-TNF access criteria also varies: elevated acute phase reactants such as CRP or ESR are present in only approximately 40–50% of patients with AS [6], while sacroiilitis visible on MRI appears to be present in less than half of patients with nr-axSpA [7, 8]. Currently, there is a lack of evidence to indicate how many SpA patients possess the unique combinations of clinical characteristics demanded by different sets of anti-TNF criteria across various settings. However, it is clear that by requiring anti-TNF users to meet clinical criteria present in only a portion of SpA patients, fewer individuals will be treated with anti-TNF therapy than if it were available to all. Importantly, the burden of SpA in terms of disease activity and impairment to be comparable among AS and nr-axSpA patients [9–11], indicating the need to treat both populations.

By limiting the number of patients treated, anti-TNF access criteria may be seen as a means of reducing the total budget impact [12] of anti-TNF agents. However, the cost-effectiveness of limiting anti-TNF therapy to patients meeting any particular set of clinical criteria has not been demonstrated. To date, some attention has been paid to the relative cost-effectiveness of anti-TNF agents in AS patients versus nr-axSpA patients [1], although the results are considered inconclusive. This is due in part to heterogeneity in the probability and magnitude of anti-TNF response observed across the small number of anti-TNF trials in nr-axSpA [13–17], which, notably, have included patients with different clinical characteristics. Although a meta-analysis indicates a slightly lower effect of anti-TNF therapy in the nr-axSpA population compared to AS [1], evidence from certain trials that have included both populations suggests the effect may be the same if patients are similar in terms of CRP levels, human leukocyte antigen (HLA)-B27 positivity, and presence of MRI inflammation [17, 18]. Unique combinations of clinical characteristics, such as those cited in anti-TNF access criteria, have not been studied in terms of their influence on the estimated cost-effectiveness of anti-TNF therapy.

The DESIR cohort is a longitudinal study of early SpA that provides clinical and cost data on a clinically heterogeneous population of both AS and nr-axSpA patients in France. Our objective was to explore how many DESIR patients would possess the unique clinical characteristics required to receive anti-TNF therapy in select settings and to examine costs and health outcomes in each of these subsets of patients. We then aimed to estimate anti-TNF cost-effectiveness over one year within each subset, with the goal of determining whether the current French restrictions on anti-TNF access [19] are the most cost-effective in that setting relative to other potential restrictions.

## Methods

### Study setting and data source

The current study was an analysis of data from the DESIR cohort, a 10-year prospective study of 708 early SpA patients recruited from 25 centres across France between October 2007 and April 2010 [20]. The DESIR cohort is a clinically heterogeneous SpA population whose characteristics have been extensively described [21–23]. At study entry, all patients were aged 18–50 and had symptoms of inflammatory back pain [24, 25] that had lasted >3 months and <3 years and was suggestive of SpA according to a rheumatologist’s assessment. Follow-up visits occurred every 6 months in the first 2 years and every year thereafter. Data from the first 3 years of DESIR follow-up, i.e., baseline visit (n = 708) plus follow-up visits at months 6 (n = 704), 12 (n = 698), 18 (n = 691), 24 (n = 692), 36 (n = 631) were available for this analysis.

The DESIR database contains clinical, quality of life, and cost data. The clinical data include many of the parameters commonly cited in access criteria for anti-TNF agents [4], including diagnosis; disease activity according to the Bath Ankylosing Spondylitis Disease Activity Index (BASDAI) and Physician Global Assessment (PhGA); X-ray, magnetic resonance imaging (MRI), and computerized tomography (CT) findings; acute phase reactants (e.g., CRP); and treatment history. The quality of life data collected in DESIR is derived from the Short Form 36 Health Survey (SF-36).

The DESIR cost data were derived from a recent cost-of-illness study, for which detailed costing methods, unit costs, and data sources have been described [26]. In summary, costing was conducted from a limited societal perspective, including all-cause direct medical costs (i.e., health resource use) and indirect costs (i.e., productivity losses), but excluding direct non-medical costs (e.g., transportation, devices, caregiver expenses), and expressed in 2013 Euros. Direct medical costs were grouped into health practitioner visits, hospitalizations including emergency room visits and surgeries, medical workups, and medications. Total direct non-medical costs were calculated as the reported number of consumed units of each cost component, multiplied by the corresponding unit cost, and summed across all categories and patients. Indirect costs were valued by multiplying the reported number of work days lost by a daily estimated wage per patient in 2013 Euros, which was based on reported professional category and average population wage data [27]. The age and sex distribution of DESIR the cohort was compared to that of the population of French workers from which average population wages were obtained and wages were not further adjusted for age and sex. Missing cost and clinical data were imputed using Monte Carlo Markov Chain multiple imputation, last observation carried forward, probabilistic imputation, or with negative values based on clinical expertise, as appropriate [26].

### Selection of anti-TNF access criteria

Most patient characteristics cited in anti-TNF access criteria [4] are routinely collected in clinical practice for multiple purposes. Using DESIR clinical data, it is possible to assess patient satisfaction of the anti-TNF access criteria in place in France [19] and numerous other settings. For the purpose of the analysis, we sought to select a practical number of sets of access criteria with clinically meaningful differences between them and the French criteria, i.e., sets citing different markers of disease severity whose prevalence would vary within the DESIR cohort. The selection process was undertaken by the research team, which included a rheumatologist (BF), epidemiologist (SH), and biostatistician (DG) with knowledge of the DESIR cohort and database. By consensus, four sets of access criteria were selected, including those from Canada [28], Germany [29] Hong Kong [30], and the United Kingdom (UK) [31]. Based on their respective criteria, these sets were anticipated to result in multiple, distinct (though potentially overlapping) subsets of DESIR patients defined as eligible for anti-TNF therapy.

### Creation of ‘study population’ datasets

We created five separate datasets containing the DESIR patients who satisfied the diagnosis and disease severity criteria for anti-TNF access in France [19], Canada [28], Germany [29], Hong Kong [30], and UK [31], respectively. These datasets were created to represent five separate ‘study populations’ of patients, each comprised of anti-TNF users and non-users who satisfied the same set of anti-TNF access criteria. As patients could satisfy multiple criteria sets, unique patients could appear in more than one study population dataset. However, as the five study population datasets were separate, only anti-TNF users and non-users who satisfied the same criteria could be compared to each other. This was done to help limit confounding by indication, as patients satisfying the same anti-TNF access criteria have comparable disease severity on a number of specific measures. Satisfaction of the treatment failure criterion, i.e., insufficient response to non-steroidal anti-inflammatory drugs (NSAIDs), was assumed for all patients.

In creating the five study population datasets, specific rules were applied in a basecase analysis and subsequently varied in sensitivity analyses. In all analyses, patients were required to satisfy the relevant criteria set no later than month 24. In the basecase analysis, anti-TNF use (yes/no) was defined based on the patient’s experience in the 1 year following the date of criteria satisfaction, which was taken as the index date for all patients. In the sensitivity analyses, anti-TNF use (yes/no) was defined over the entire study period, with date of criteria satisfaction taken as the index date for anti-TNF non-users and date of anti-TNF initiation taken as the index date for anti-TNF users. In all analyses, outcomes were observed in the 1 year following the index date. Because the start point for the 1 year observation period was defined differently in the basecase and sensitivity analyses, patients who were classed as anti-TNF non-users in the basecase analysis could be classed as anti-TNF users in the sensitivity analyses.

To be included in the basecase analysis, anti-TNF users were required not to have initiated anti-TNF therapy prior to criteria satisfaction (rule 1). Anti-TNF users were further required to have initiated the anti-TNF <6 months after criteria satisfaction (rule 2). Consequently, anti-TNF users who initiated anti-TNF before criteria satisfaction or >6 months after criteria satisfaction were excluded from the basecase analysis. These rules were applied in order to include only anti-TNF users with a similar length of anti-TNF exposure in the basecase analysis, in which outcomes were observed following the date of criteria satisfaction rather than therapy initiation.

In the first sensitivity analysis, anti-TNF users were permitted to receive the anti-TNF prior to criteria satisfaction (rule 1 lifted). In the second sensitivity analysis, anti-TNF users were permitted to receive the anti-TNF >6 months after criteria satisfaction (rule 2 lifted). In the third sensitivity analysis, anti-TNF users were permitted to receive the anti-TNF at any time point (rules 1 and 2 lifted). A separate sensitivity analysis was conducted to explore the impact of simulating a 24-week stopping rule for anti-TNF non-responders, defined as patients who did not achieve a 50% relative change or absolute change of 2 on the BASDAI scale [32] one visit post-therapy initiation. In this analysis, anti-TNF costs accumulated by non-responders after 24 weeks of therapy were excluded. Additional sensitivity analyses were conducted to explore the impact of excluding indirect costs in all scenarios.

### Descriptive statistics

Sociodemographic and clinical characteristics at baseline and at time of criteria satisfaction among patients in each of the five basecase study population datasets were described in terms of mean (SD) and frequency (%) as appropriate. Descriptive statistics were also produced to describe the number of anti-TNF users in the DESIR cohort who did not satisfy any of the selected criteria sets (and were therefore excluded from analysis) as well as the number of anti-TNF non-responders in the basecase study population datasets and their total time on anti-TNF therapy.

### Adjustment of costs and QALYs

To control for differences between anti-TNF users and non-users, we used linear regression models to estimate adjusted total costs (i.e., direct medical plus indirect costs) in the 1 year post-index. Independent variables considered to be potential confounders of the relationship between anti-TNF use and costs were first tested in univariate models of costs, specifically age, sex, education, marital status, disease duration, smoking (yes vs. no/do not know), HLA-B27 status and presence of peripheral arthritis at baseline, and the Bath Ankylosing Spondylitis Functional Index (BASFI), BASDAI, PhGA, CRP, and SF-36 at the patient’s index date. The same variables were then each tested in preliminary multivariate models of costs that included BASFI (the strongest predictor of costs in univariate analyses) and anti-TNF use. Independent variables that changed the coefficient for anti-TNF use by more than 10% in the preliminary multivariate models were included in the final multivariate costs model.

Total QALYs in the one year post-index were calculated using SF6D utility weights derived from SF-36 health states, following the area under the curve method [33, 34]. Again to control for differences between anti-TNF users and non-users, we used linear regression models to derive adjusted mean QALYs. Independent variables as above were first tested in univariate models then in preliminary multivariate models that included SF-36 at time of criteria satisfaction (the strongest predictor of QALY in univariate analyses) and anti-TNF use. Independent variables that changed the coefficient for anti-TNF use by more than 10% in preliminary multivariate models were included in the final multivariate QALY model.

### Cost-effectiveness analysis using adjusted costs and QALYs

For each of the five study population datasets, we calculated the incremental cost-effectiveness ratio (ICER) comparing the costs and QALYs of anti-TNF users versus non-users, i.e., the incremental cost per additional QALY gained by treating with an anti-TNF, using the standard formula: [(Cost anti-TNF- Cost no anti-TNF)/(QALYs anti-TNF- QALYs no anti-TNF)]. To explore the range of uncertainty around mean costs and QALYs, we used non-parametric bootstrapping [35], repeating the same procedures for each study population datasets (i.e., group of patients satisfying a given criteria set). Specifically, 10,000 bootstrap samples were generated (i.e., by sampling with replacement), stratified by anti-TNF users and non-users. For each bootstrapped sample, linear regression models were fitted for costs and QALYs; although the models were fitted separately, the data used were from the same samples, meaning the interdependence of costs and QALYs was accounted for. Adjusted mean costs and QALYs and hence the incremental costs and QALYs were then estimated from the models. The 2.5th and 97.5th percentiles of the bootstrapped distribution were used to estimate 95% confidence intervals (CI) for the incremental costs and QALYs.

## Results

Table 1 shows the diagnosis and disease severity criteria for anti-TNF access in France, Canada, Germany, Hong Kong, and the UK, as well as the number of DESIR patients who satisfied the respective sets. The criteria sets from the UK and Hong Kong both required a diagnosis of AS, while those from Canada, France, and Germany were inclusive of nr-axSpA patients. Anti-TNF access criteria from France were satisfied by the largest number of DESIR patients (197/708; 27.8%), followed by Germany (175/708; 25.1%), Canada (169/708; 23.8%), the UK (86/708;12.1%) and Hong Kong (61/708; 8.6%).

**Table 1:** Selected criteria sets and satisfaction at baseline among 708 DESIR patients

Criteria set origin	Patients ever satisfying criteria set (n) and percent of total (N = 708)	Diagnosis and disease severity criteria
France	n = 197 (27.8%)	BASDAI ≥4 New York criteria for AS OR involvement of SJ OR spine by X-ray, CT or MRI Physician’s Global Assessment ≥4
Germany	n = 175 (24.7%)	BASDAI ≥4 ASAS criteria Positive MRI or Elevated CRP^b^ (i.e., minimum 1 of 2 criteria)
Canada	n = 169 (23.8%)	BASDAI ≥4 Sacroiilitis or spinal inflammation on X-ray, CT or MRI Elevated CRP or ESR and/or Inflammatory lesions in the sacroiliac joints and/or spine on MRI and/or Expert opinion^a^ (Minimum 2 of 3 criteria)
United Kingdom	n = 86 (12.1%)	BASDAI ≥4 Modified New York criteria for AS Spinal pain in last week 4/10 VAS
Hong Kong	n = 61 (8.6%)	BASDAI ≥4 Modified New York criteria for AS Morning stiffness ≥45 min Inflammatory back pain ≥40/100 VAS Patient Global Assessment ≥40/100 VAS

*CT* computerized tomography, *MRI* magnetic resonance imaging, *BASDAI* Bath Ankylosing Spondylitis Disease Activity Index, *AS* ankylosing spondylitis, *CRP* C-reactive protein, *ESR* erythrocyte sedimentation rate, *SJ* sacroiliac joint, *VAS* visual analogue scale

^a^Positive expert opinion defined in the analysis as Physician’s Global Assessment ≥4

^b^NA at baseline, values reported for month 6

Table 2 shows the characteristics of anti-TNF users and non-users in each of the basecase study population datasets. The proportion of anti-TNF users was highest among patients who met the Hong Kong criteria (32/61; 52.5%), followed by the UK (40/86; 46.5%), Canada (71/169; 42.0%), France (80/197; 40.6%), and Germany (67/175; 38.3%). Among a total 225 anti-TNF users in the DESIR cohort, 107 (47.6%) never satisfied the French anti-TNF access criteria, while 94 (41.8%) never satisfied any of the selected criteria sets and were thus excluded from the analysis. The characteristics of excluded anti-TNF users are shown in Additional file 1: Table S1.

**Table 2:** Characteristics of DESIR patients satisfying selected criteria sets

	Canada n = 169		France n = 197		UK n = 86		Germany n = 175		Hong Kong n = 61	
	Anti-TNF non-user (n = 98)	Anti-TNF user (n = 71)	Anti-TNF non-user (n = 117)	Anti-TNF user (n = 80)	Anti-TNF non-user (n = 46)	Anti-TNF user (n = 40)	Anti-TNF non-user (n = 108)	Anti-TNF user (n = 67)	Anti-TNF non-user (n = 29)	Anti-TNF user (n = 32)
At baseline										
Age	32.8 ± 8.5	33.5 ± 9.3	33.2 ± 8.7	33 ± 9.1	33 ± 7.6	32.6 ± 9.6	32.7 ± 7.9	33.1 ± 9.4	35 ± 8	31.8 ± 8.5
Male	53 (54.1%)	40 (56.3%)	58 (49.6%)	59 (53.8%)	27 (58.7%)	24 (60%)	59 (54.6%)	37 (55.2%)	17 (58.6%)	19 (59.4%)
Post-secondary education	55 (56.1%)	42 (59.2%)	65 (55.6%)	48 (60%)	22 (47.8%)	20 (50%)	60 (55.6%)	35 (59.7%)	11 (37.9%)	14 (43.8%)
Married	58 (59.2%)	40 (59.2%)	71 (60.7%)	48 (60%)	24 (52.2%)	23 (57.5%)	69 (63.9%)	40 (59.7%)	17 (58.6%)	19 (59.4%)
Smoking	41 (41.8%)	36 (50.7%)	50 (42.7%)	38 (47.5%)	23 (50.0%)	22 (55.0%)	44 (40.7%)	35 (52.2%)	12 (41.4%)	19 (59.4%)
Disease duration	1.5 ± 0.9	1.6 ± 0.8	1.6 ± 0.9	1.6 ± 0.8	1.7 ± 0.9	1.6 ± 0.8	1.5 ± 0.9	1.6 ± 0.8	1.7 ± 0.9	1.6 ± 0.8
Peripheral Arthritis	54 (55.1%)	49 (69%)	65 (55.6%)	55 (68.8%)	22 (47.8%)	25 (62.5%)	56 (51.9%)	46 (68.7%)	13 (44.8%)	21 (65.6%)
HLA-B27: positive	59 (60.2%)	44 (62%)	66 (56.4%)	48 (60%)	32 (69.6%)	25 (62.5%)	83 (76.9%)	46 (68.7%)	18 (62.1%)	19 (59.4%)
Radiographic sacroiliitis	42 (42.9%)	40 (56.3%)	49 (41.9%)	44 (55.0%)	28 (60.9%)	30 (75.0%)	49 (45.4%)	41 (61.2%)	15 (51.7%)	25 (78.1%)
Sacroiilitis or spine inflammation on MRI	67 (68.4%)	49 (69.0%)	67 (57.3%)	49 (61.3%)	25 (54.3%)	26 (65.0%)	80 (74.1%)	52 (77.6%)	12 (41.4%)	21 (65.6%)
BASDAI ≥ 4	74 (75.5%)	67 (94.4%)	91 (77.8%)	76 (95.0%)	34 (73.9%)	39 (97.5%)	78 (72.2%)	62 (92.5%)	21 (72.4%)	31 (96.9%)
CRP > 10 mg/L	24 (24.5%)	31 (43.7%)	25 (21.4%)	33 (41.3%)	8 (17.4%)	21 (52.5%)	26 (24.1%)	32 (47.8%)	5 (17.2%)	14 (43.8%)
Physician’s global assessment ≥4	76 (77.6%)	66 (93.0%)	92 (78.6%)	75 (93.8%)	33 (71.7%)	38 (95.0%)	73 (67.6%)	61 (91.0%)	20 (69.0%)	31 (96.9%)
Inflammatory back pain ≥ 4	79 (80.6%)	68 (95.8%)	96 (82.1%)	76 (95.0%)	40 (87.0%)	40 (100%)	88 (81.5%)	64 (95.5%)	25 (86.2%)	32 (100%)
Morning stiffness ≥45 min	53 (54.1%)	47 (66.2%)	62 (53.0%)	56 (70.0%)	20 (43.5%)	31 (77.5%)	57 (52.8%)	47 (70.1%)	18 (62.1%)	30 (93.8%)
Patient global assessment ≥4	73 (74.5%)	65 (91.5%)	88 (75.2%)	74 (92.5%)	33 (71.7%)	38 (95.0%)	79 (73.1%)	60 (89.6%)	23 (79.3%)	31 (96.9%)
At criteria satisfaction										
BASDAI	56.2 ± 12.7	60.6 ± 11.4	55.4 ± 12.2	60.9 ± 10.9	52.8 ± 10.5	58.9 ± 11.9	55.5 ± 12.7	58.7 ± 10.8	56.1 ± 12	59.3 ± 11.7
BASFI	35.8 ± 21.8	43.9 ± 20.2	35.9 ± 21.5	45 ± 19.8	30.8 ± 20	47.4 ± 20.8	36.5 ± 23.5	42.1 ± 19.7	39 ± 21.6	46.5 ± 22
SF-36	0.6 ± 0.1	0.5 ± 0.1	0.6 ± 0.1	0.5 ± 0.1	0.6 ± 0.1	0.5 ± 0.1	0.6 ± 0.1	0.5 ± 0.1	0.6 ± 0.1	0.5 ± 0.1
Physician’s global assessment	5.3 ± 1.2	6.7 ± 1.6	5.3 ± 1.2	6.8 ± 1.5	4.7 ± 1.7	6.8 ± 1.7	4.5 ± 1.8	6.4 ± 2	5 ± 1.6	6.7 ± 1.6
CRP	10.9 ± 18.6	20.1 ± 24.6	9.5 ± 17.2	18.8 ± 23.8	7.7 ± 9.3	22.3 ± 25.5	10.9 ± 12.5	19.7 ± 23.7	7.6 ± 10.2	15.8 ± 20.6

Statistics presented are: Mean ± SD, or N (%)

**Fig. 1:**
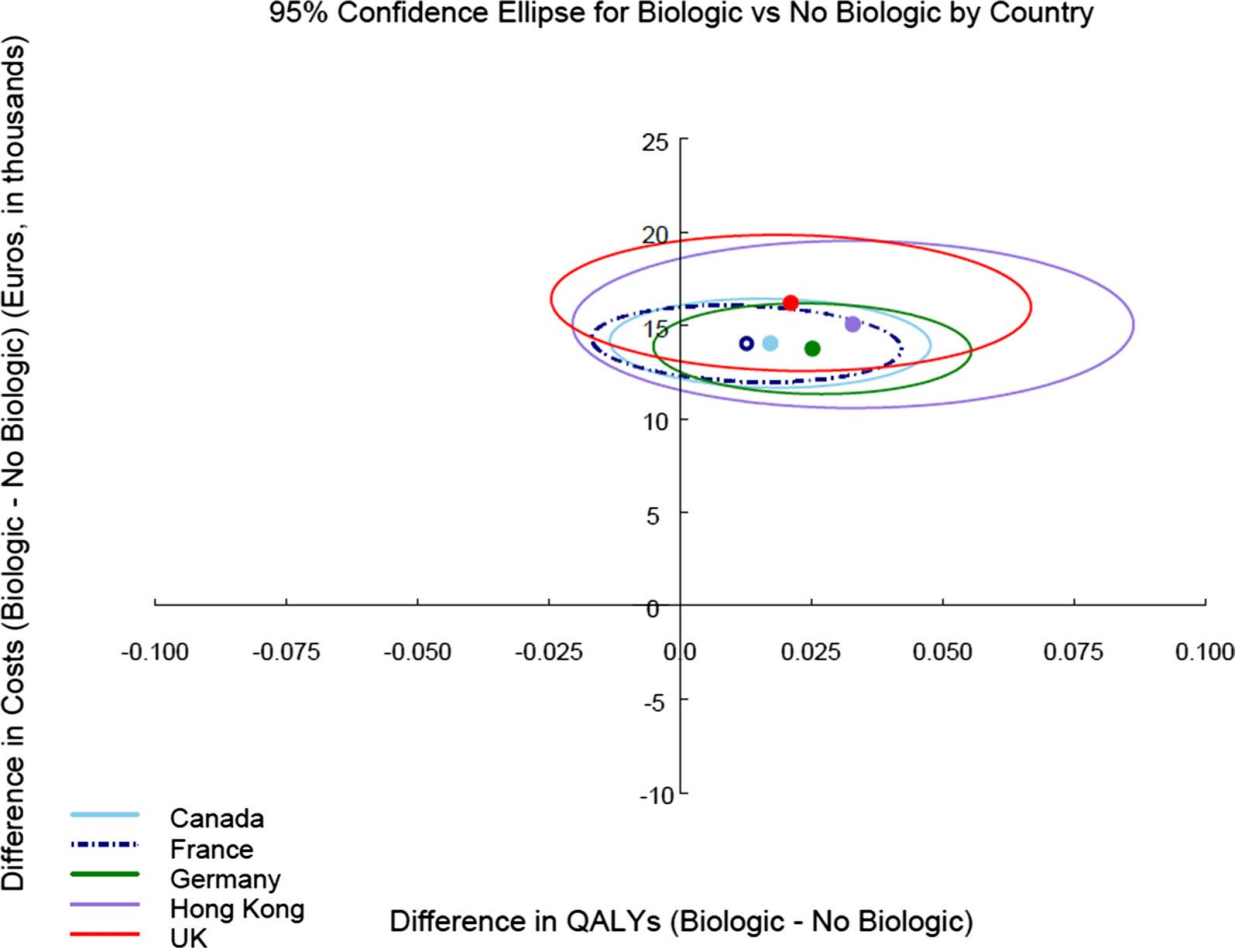
Confidence intervals around ICERs from each of the five study populations

Table 3 shows the unadjusted and adjusted costs of patients in the five basecase study population datasets. In final multivariate models, costs were adjusted for smoking and HLA-B27 status at baseline, and BASFI, PhGA, and CRP at date of criteria satisfaction; QALYs were adjusted for age, sex, education, smoking, HLA-B27 status and peripheral arthritis at baseline, and SF-36, PhGA, and CRP at date of criteria satisfaction. Table 4 shows the incremental costs and QALYs and ICERs over one year for each of the five study populations in the basecase analysis. The most favourable cost-effectiveness point estimate was derived from the study population satisfying the Hong Kong criteria (ICER €456,850), followed by Germany (€545,808), the UK (€766,217), and Canada (€818,186). The highest ICER was derived from the study population satisfying the French criteria (€1,105,859) However, as shown in Fig. 1, the confidence intervals surrounding the point estimates for the incremental costs and QALYs derived from each of the five study populations were overlapping, indicating uncertainty in the results of the analysis.

**Table 3:** Unadjusted and adjusted costs, SF6D utility scores and QALYs among DESIR patients satisfying selected criteria sets

	Canada n = 169		France n = 197		UK n = 86		Germany n = 175		Hong Kong n = 61	
	Anti-TNF non-user (n = 98)	Anti-TNF user (n = 71)	Anti-TNF non-user (n = 117)	Anti-TNF user (n = 80)	Anti-TNF non-user (n = 46)	Anti-TNF user (n = 40)	Anti-TNF non-user (n = 108)	Anti-TNF user (n = 67)	Anti-TNF non-user (n = 29)	Anti-TNF user (n = 32)
Unadjusted										
Total costs (Mean ± SD)	1981 ± 2812	15,448 ± 6155	2117 ± 2976	15,311 ± 5801	1426 ± 1852	16,475 ± 7506	2044 ± 3351	15,339 ± 6453	1697 ± 2447	15,132 ± 6548
Health Practitioner	655 ± 745	896 ± 772	640 ± 720	1000 ± 939	440 ± 573	971 ± 1034	605 ± 750	953 ± 1013	472 ± 722	835 ± 682
Hospital	532 ± 1289	710 ± 2010	615 ± 1505	678 ± 1913	530 ± 1326	646 ± 1394	454 ± 1090	731 ± 2036	695 ± 1541	591 ± 1184
Medical Act	178 ± 238	399 ± 362	185 ± 247	396 ± 352	155 ± 239	449 ± 417	154 ± 228	386 ± 313	162 ± 330	433 ± 388
Medication	149 ± 135	12,511 ± 5252	143 ± 132	12,364 ± 5114	126 ± 100	12,742 ± 5844	145 ± 125	12,409 ± 5497	138 ± 120	11,981 ± 4977
Productivity	467 ± 1893	932 ± 2796	534 ± 1987	872 ± 2411	176 ± 526	1667 ± 4059	687 ± 2661	859 ± 2776	231 ± 672	1292 ± 3271
Total costs: Med (IQR)	913 (362, 2343)	14,172 (12,741, 17,495)	920 (364, 2343)	14,293 (12,806, 17,537)	839 (326, 1621)	14,963 (13,110, 18,742)	865 (323, 2092)	14,111 (12,825, 16,356)	809 (298, 1681)	14,404 (12,579, 17,222)
SF6D (Mean ± SD)										
Index	0.579 ± 0.101	0.532 ± 0.078	0.579 ± 0.098	0.533 ± 0.075	0.602 ± 0.088	0.528 ± 0.074	0.594 ± 0.105	0.536 ± 0.079	0.594 ± 0.078	0.531 ± 0.073
6 M after index visit	0.645 ± 0.108	0.644 ± 0.130	0.638 ± 0.105	0.635 ± 0.128	0.644 ± 0.103	0.630 ± 0.130	0.650 ± 0.111	0.652 ± 0.132	0.609 ± 0.094	0.624 ± 0.128
12 M after INDEX VISit	0.647 ± 0.118	0.620 ± 0.123	0.639 ± 0.115	0.612 ± 0.122	0.639 ± 0.108	0.609 ± 0.112	0.646 ± 0.124	0.632 ± 0.123	0.633 ± 0.099	0.595 ± 0.107
Total QALY (Mean ± SD)	0.63 ± 0.09	0.61 ± 0.10	0.62 ± 0.09	0.60 ± 0.10	0.63 ± 0.08	0.60 ± 0.10	0.63 ± 0.09	0.62 ± 0.10	0.61 ± 0.07	0.59 ± 0.09
Adjusted (Mean ± SE)										
Total costs	1703 ± 366	15,741 ± 802	1763 ± 347	15,773 ± 687	756 ± 644	16,952 ± 1160	1874 ± 408	15,617 ± 852	722 ± 946	15,764 ± 1229
Total QALY	0.61 ± 0.01	0.63 ± 0.01	0.61 ± 0.01	0.62 ± 0.01	0.61 ± 0.01	0.63 ± 0.02	0.62 ± 0.01	0.64 ± 0.01	0.59 ± 0.02	0.62 ± 0.02

**Table 4:** Comparative estimates of costs, QALYs, and ICERs: basecase analysis

	Anti-TNF user	Anti-TNF non-user	Increment (user vs non-user)	95% CI^a^
Canada				
Costs	15,741	1703	14,038	(12,179, 15,991)
QALYs	0.626	0.609	0.017	(−0.008, 0.042)
ICER			818,186	(330,082, Dominated)
France				
Costs	15,773	1763	14,010	(12,423, 15,694)
QALYs	0.620	0.607	0.013	(−0.011, 0.036)
ICER			1,105,859	(375,227, Dominated)
UK				
Costs	16,952	756	16,195	(13,327, 19,181)
QALYs	0.627	0.606	0.021	(−0.015, 0.059)
ICER			766,217	(264,164, Dominated)
Germany				
Costs	15,617	1874	13,743	(11,848, 15,759)
QALYs	0.642	0.617	0.025	(0.001, 0.050)
ICER			545,808	(272,286, 18,727,278)
Hong Kong				
Costs	15,764	722	15,042	(11,825, 18,898)
QALYs	0.619	0.586	0.033	(−0.011, 0.076)
ICER			456,850	(189,636, Dominated)

^a^Lower bound = 2.5th, upper bound = 97.5th percentile of bootstrapped distribution

A positive anti-TNF response one visit post-therapy initiation was achieved by approximately half of anti-TNF users who satisfied the criteria from Canada (n = 39; 54.9%), France (n = 42; 52.5%), and Germany (35; 51.5%), respectively, and by approximately forty percent of anti-TNF users who satisfied criteria from the UK (n = 17; 42.5%) and Hong Kong (n = 13; 40.6%). In each of the five study populations, 90% or more of non-responders continued anti-TNF therapy for one or more years (Additional file 2: Table S2). In the sensitivity analysis that examined the effect of excluding costs accumulated past 24 weeks by anti-TNF non-responders, the incremental cost per QALY was reduced by approximately 25% (France: €857,992 vs. €1,105,859; Canada: € 626,459 vs. €818,186; Germany: € 422,568 vs. €545,808); UK €578,899 vs. €766,217; Hong Kong €335,418 vs. €456,850) (Table 5). Consistent with this finding, utility gain was observed to be lower among anti-TNF non-responders compared to responders (Table 6).

**Table 5:** Comparative estimates of costs, QALYs, and ICERs: sensitivity analysis excluding non-responder anti-TNF costs past 24 weeks

	Anti-TNF user	Anti-TNF non-user	Increment (user vs non-user)	95% CI^a^
Canada				
Costs	12,405	1656	10,749	(8868, 12,789)
QALYs	0.626	0.609	0.017	(−0.008, 0.042)
ICER			626,459	(247,149, Dominated)
France				
Costs	12,566	1696	10,870	(9144, 12,751)
QALYs	0.620	0.607	0.013	(−0.011, 0.036)
ICER			857,992	(284,242, Dominated)
UK				
Costs	12,973	737	12,236	(8981, 15,769)
QALYs	0.627	0.606	0.021	(−0.015, 0.059)
ICER			578,899	(187,442, Dominated)
Germany				
Costs	12,321	1682	10,640	−877,112,728
QALYs	0.642	0.617	0.025	(0.001, 0.050)
ICER			422,568	(206,749, 14,587,057)
Hong Kong				
Costs	11,935	891	11,044	(7243, 15,470)
QALYs	0.619	0.586	0.033	(−0.011, 0.076)
ICER			335,418	(124,073, Dominated)

^a^Lower bound = 2.5 th, upper bound = 97.5 th percentile of bootstrapped distribution

**Table 6:** Utility gain 6 and 12 months post-therapy initiation in anti-TNF responders and non-responders

	Canada	France	UK	Germany	HK
Unadjusted (Mean ± SD)					
All users					
Index	0.53 ± 0.08	0.53 ± 0.08	0.53 ± 0.07	0.54 ± 0.08	0.53 ± 0.07
6 M after index visit	0.64 ± 0.13	0.63 ± 0.13	0.63 ± 0.13	0.65 ± 0.13	0.62 ± 0.13
12 M after index visit	0.62 ± 0.12	0.61 ± 0.12	0.61 ± 0.11	0.63 ± 0.12	0.60 ± 0.11
Responders					
Index	0.55 ± 0.09	0.55 ± 0.09	0.53 ± 0.08	0.55 ± 0.09	0.55 ± 0.08
6 M after index visit	0.68 ± 0.12	0.67 ± 0.12	0.65 ± 0.12	0.69 ± 0.12	0.67 ± 0.11
12 M after index visit	0.65 ± 0.12	0.64 ± 0.12	0.62 ± 0.10	0.66 ± 0.11	0.60 ± 0.10
Non-responders					
Index	0.52 ± 0.06	0.52 ± 0.06	0.52 ± 0.07	0.52 ± 0.06	0.52 ± 0.07
6 M after index visit	0.60 ± 0.13	0.59 ± 0.12	0.62 ± 0.14	0.61 ± 0.13	0.59 ± 0.13
12 M after index visit	0.59 ± 0.12	0.58 ± 0.12	0.61 ± 0.12	0.60 ± 0.13	0.59 ± 0.11

In the sensitivity analysis using the basecase study population, but excluding indirect costs, all ICERs became more favourable (Additional file 3: Table S3). In all additional sensitivity analyses, i.e., including anti-TNF users who initiated therapy prior to and/or 6–12 months after criteria satisfaction, anti-TNF agents were dominated in all study populations (Additional file 3: Table S3); this finding did not change upon the exclusion of indirect costs (data not shown).

## Discussion

To our knowledge, this is the first study to explore what proportion of SpA patients in a single cohort possesses the unique combination of clinical characteristics demanded by select sets of anti-TNF access criteria. We found that the proportion of DESIR patients eligible to receive anti-TNF therapy ranged from 9 to 28%, depending on the criteria set considered. For illustrative purposes, we note that assuming a SpA prevalence of 0.43% in France [36], this may translate to as few as 39 or as many as 120 people per 100,000 population per year being recommended anti-TNF therapy. At an estimated cost of €13,000 for a full year of anti-TNF therapy [26], the additional 81 people treated under the less restrictive access conditions would have an annual budget impact of €1.05 million. One of the contributions of the present study is in highlighting the potential role of anti-TNF access criteria, as at the current cost of anti-TNF therapy even a small number of additional patients treated will correspond to a large increase in health budgets, which may or may not represent good value for the public.

This study focused on the comparative cost-effectiveness of selected criteria sets in the French setting, and the absolute ICER values generated here should be interpreted with caution. The ICERs were produced using data over a single year using real-world data, and it should be stressed that these cannot be compared to ICERs from models that employ a lifetime horizon, estimate treatment effectiveness using RCT data, or assume that non-responders will be withdrawn from treatment. Lifetime cost-effectiveness models have the important capability of acknowledging that not all benefits of anti-TNF therapy will be realized within a short time frame; in general, anti-TNF agents appear more cost-effective in models with longer time horizons [37]. Recently, the UK’s National Institute for Health and Clinical Excellence (NICE) [1] reported upperbound ICERs of £66,529 per QALY for AS patients and £34,232 per QALY for nr-axSpA patients based on lifetime cost-effectiveness models. These are vastly more favourable than the ICERs estimated here, reflecting, in part, the impact of including the latent benefits of anti-TNF therapy. However, including these predicted benefits required extrapolating outcomes beyond periods for which observed data are available. In contrast, the present analysis has provided important observed data on the costs and benefits associated with anti-TNF use in a real-world setting. Importantly, the NICE models assume that all non-responders will discontinue therapy at 12-weeks [1], yet we found that the vast majority of non-responders continued therapy for a year or more. The continuation of therapy among non-responders appears to be one reason that ICERs estimated in the basecase analysis here are less favourable than those estimated by the NICE: in a sensitivity analysis, we found that ICERs were reduced by approximately 25% by simulating a 24-week stopping rule. As well, it may be noted that we found only modest QALY gains associated with anti-TNF use over 1 year, though utility gains were up to 0.03 units higher among anti-TNF responders compared to non-responders. Few studies have directly reported utility gain associated with anti-TNF use, and the NICE cost-effectiveness models predicted utility from BASDAI and BASFI scores using an algorithm that has not been externally evaluated [1]. It is difficult to determine whether the utility gain associated with anti-TNF use among DESIR patients is similar to what was predicted by the NICE, and there is an outstanding need for studies to report observed utility gain among patients using anti-TNF therapy.

In this study, we were unable to confirm whether France’s restrictions are the most cost-effective in that setting relative to other potential restrictions over the short term; the uncertainty around the results in the basecase analysis indicates all of the criteria sets compared here may be equally cost-effective. However, the study makes a number of observations that highlight the potential for anti-TNF access regulations to shape the therapy’s cost-effectiveness, in part by defining the target population for initiation, which both influences the likelihood of anti-TNF response and determines the appropriate population of non-users for comparison. In this study, we found that between 41% and 55% of anti-TNF users across the five study population datasets achieved a BASDAI 50 response, and the mean SF6D utility gain one year following anti-TNF initiation was higher among responders compared to non-responders. At the same time, cost-effectiveness estimates did not vary directly in accordance with the proportion of BASDAI 50 responders: the most favourable ICER was derived from the Hong Kong criteria dataset, though it had the lowest proportion of responders. This discrepancy appears to result from the lower utility among the anti-TNF non-users in the Hong Kong criteria dataset, which translated to a larger incremental difference in QALYs compared to other study population datasets. These findings underscore that, to maximize cost-effectiveness, anti-TNF therapy must be directed to patients mostly likely to experience substantial improvement in quality of life when compared to conventional care, and there is a strong need to inform anti-TNF access criteria with evidence to characterize this patient population. To date, a good deal of research has demonstrated predictors of anti-TNF response, both among AS patients [38–41] and nr-axSpA patients [9, 13–15, 42]. However, there are shortcomings in this evidence base, with more data derived from RCT populations [13–15, 18, 43] than observational cohorts [9, 38, 39, 42] and more evidence on certain markers (e.g., CRP [15, 39, 43]) than others (e.g., HLA-B27 [42]). Furthermore, few studies have modelled anti-TNF response based on combinations of clinical characteristics, which should be more useful for decision-making than single predictors [41]. Perhaps most importantly, most studies have defined anti-TNF response in binary terms using clinical measures such as the BASDAI [32], ASAS40 [44], or ASDAS [45] and the effect on quality of life of achieving a response as defined by these measures has not been established [46].

The results of this study suggest that one crucial strategy to improve anti-TNF cost-effectiveness is to ensure treatment discontinuation by anti-TNF users not experiencing clinical benefit. To implement this strategy, it would be useful to confirm minimally important differences on common quality of life measures [47, 48], to encourage clinicians to measure the benefits of anti-TNF therapy in terms of quality of life, and to help patients and providers engage in a shared decision-making process around discontinuation. We acknowledge that enforcement of regulations surrounding anti-TNF therapy is challenging, as reflected by the fact that 40% of anti-TNF users in DESIR did not satisfy the French anti-TNF access criteria. However, the potential for anti-TNF access criteria to shape the cost-effective use of these agents should not be ignored, and the rationale for initiating therapy only among patients likely to benefit- and for discontinuing therapy when appropriate- should be known by patients and providers.

Certain limitations of this study should be noted. For one, results differed depending on which anti-TNF users were included in the analysis: when anti-TNF users who received therapy prior to criteria satisfaction or 6–12 months after criteria satisfaction were included, anti-TNF therapy was dominated in all scenarios. This finding points to a possible role for timing of anti-TNF initiation in determining the therapy’s cost-effectiveness; however, the results could also be explained by unmeasured, time-variant confounders. In general, the data analyzed here were derived from a non-randomized study, meaning all results are subject to confounding by indication. To help control for this, we made comparisons only between anti-TNF users and non-users who satisfied the same set of access criteria and we further adjusted costs and QALYs for known confounders, though residual confounding cannot be ruled out. In terms of other study limitations, we assumed that all patients met treatment failure criteria, which were defined differently in the selected criteria sets. It should be noted that up to a third of SpA patients may achieve clinical remission with NSAIDs alone [49] and anti-TNF therapy will necessarily be more cost-effective if used only by patients who have failed this less costly treatment. The present study did not evaluate the number of NSAIDs that should be tried before anti-TNF therapy in order to maximize cost-effectiveness, which is a limitation.

Despite its limitations, this study is unique in having used data from a heterogeneous, real-world population of SpA patients to demonstrate the influence of patient characteristics on anti-TNF cost-effectiveness estimates. In line with the initiative to incorporate economic evidence into clinical guidelines [50, 51], future research should focus on confirming what combination of patient characteristics best predicts quality of life improvement following anti-TNF therapy and informing anti-TNF access criteria with this evidence. As a substantial number of anti-TNF users in the DESIR cohort did not satisfy any of the selected criteria sets, and as discontinuation of anti-TNF therapy following non-response was infrequently observed, this study further calls for a discussion as to the practical application of regulations surrounding anti-TNF therapy.

## Additional files

**Additional file 1: Table S1.** Characteristics of anti-TNF users not satisfying selected criteria sets.

**Additional file 2: Table S2.** Total time on anti-TNF therapy among non-responders (from initiation until end of follow-up).

**Additional file 3: Table S3.** Comparative estimates of costs, QALYs, and ICERs in sensitivity analyses.

## References

[1] Corbett M, Soares M, Jhuti G, Rice S, Spackman E, Sideris E, Moe-Byrne T, Fox D, Marzo-Ortega H, Kay L, Woolacott N, Palmer S (2016). Tumour necrosis factor-alpha inhibitors for ankylosing spondylitis and non-radiographic axial spondyloarthritis: a systematic review and economic evaluation. Health Technol Assess..

[2] Maxwell LJ, Zochling J, Boonen A, Singh JA, Veras MM, Tanjong Ghogomu E, Benkhalti Jandu M, Tugwell P, Wells GA (2015). TNF-alpha inhibitors for ankylosing spondylitis. Cochrane Database Syst Rev..

[3] Callhoff J, Sieper J, Weiss A, Zink A, Listing J (2015). Efficacy of TNFalpha blockers in patients with ankylosing spondylitis and non-radiographic axial spondyloarthritis: a meta-analysis. Ann Rheum Dis.

[4] van den Berg R, Stanislawska-Biernat E, van der Heijde DM (2011). Comparison of recommendations for the use of anti-tumour necrosis factor therapy in ankylosing spondylitis in 23 countries worldwide. Rheumatology (Oxford).

[5] Reveille JD (2011). Epidemiology of spondyloarthritis in North America. Am J Med Sci.

[6] Reveille JD (2015). Biomarkers for diagnosis, monitoring of progression, and treatment responses in ankylosing spondylitis and axial spondyloarthritis. Clin Rheumatol.

[7] van den Berg R, de Hooge M, van Gaalen F, Reijnierse M, Huizinga T, van der Heijde D (2013). Percentage of patients with spondyloarthritis in patients referred because of chronic back pain and performance of classification criteria: experience from the Spondyloarthritis Caught Early (SPACE) cohort. Rheumatology (Oxford).

[8] Joven BE, Navarro-Compan V, Rosas J, Fernandez Dapica P, Zarco P, De Miguel E, ESPeranza working group (2017). Diagnostic value and validity of early spondyloarthritis features: results from the Esperanza Cohort. Arthritis Care Res (Hoboken)..

[9] Ciurea A, Scherer A, Exer P, Bernhard J, Dudler J, Beyeler B, Kissling R, Stekhoven D, Rufibach K, Tamborrini G, Weiss B, Muller R, Nissen MJ, Michel BA, van der Heijde D, Dougados M, Boonen A, Weber U (2013). Rheumatologists of the swiss clinical quality management program for axial spondyloarthritis. Tumor necrosis factor alpha inhibition in radiographic and nonradiographic axial spondyloarthritis: results from a large observational cohort. Arthritis Rheum.

[10] Kiltz U, Baraliakos X, Karakostas P, Igelmann M, Kalthoff L, Klink C, Krause D, Schmitz-Bortz E, Florecke M, Bollow M, Braun J (2012). Do patients with non-radiographic axial spondylarthritis differ from patients with ankylosing spondylitis?. Arthritis Care Res (Hoboken)..

[11] Rudwaleit M, Haibel H, Baraliakos X, Listing J, Marker-Hermann E, Zeidler H, Braun J, Sieper J (2009). The early disease stage in axial spondylarthritis: results from the German Spondyloarthritis inception cohort. Arthritis Rheum.

[12] Sullivan SD, Mauskopf JA, Augustovski F, Jaime Caro J, Lee KM, Minchin M, Orlewska E, Penna P, Rodriguez Barrios JM, Shau WY (2014). Budget impact analysis-principles of good practice: report of the ISPOR 2012 budget impact analysis good practice ii task force. Value Health..

[13] Dougados M, van der Heijde D, Sieper J, Braun J, Maksymowych WP, Citera G, Miceli-Richard C, Wei JC, Pedersen R, Bonin R, Rahman MU, Logeart I, Wajdula J, Koenig AS, Vlahos B, Alvarez D, Bukowski JF (2014). Symptomatic efficacy of etanercept and its effects on objective signs of inflammation in early nonradiographic axial spondyloarthritis: a multicenter, randomized, double-blind, placebo-controlled trial. Arthritis Rheumatol..

[14] Haibel H, Rudwaleit M, Listing J, Heldmann F, Wong RL, Kupper H, Braun J, Sieper J (2008). Efficacy of adalimumab in the treatment of axial spondylarthritis without radiographically defined sacroiliitis: results of a twelve-week randomized, double-blind, placebo-controlled trial followed by an open-label extension up to week fifty-two. Arthritis Rheum..

[15] Sieper J, van der Heijde D, Dougados M, Mease PJ, Maksymowych WP, Brown MA, Arora V, Pangan AL (2013). Efficacy and safety of adalimumab in patients with non-radiographic axial spondyloarthritis: results of a randomised placebo-controlled trial (ABILITY-1). Ann Rheum Dis..

[16] Barkham N, Keen HI, Coates LC, O’Connor P, Hensor E, Fraser AD, Cawkwell LS, Bennett A, McGonagle D, Emery P (2009). Clinical and imaging efficacy of infliximab in HLA-B27-positive patients with magnetic resonance imaging-determined early sacroiliitis. Arthritis Rheum..

[17] Landewe R, Braun J, Deodhar A, Dougados M, Maksymowych WP, Mease PJ, Reveille JD, Rudwaleit M, van der Heijde D, Stach C, Hoepken B, Fichtner A, Coteur G, de Longueville M, Sieper J (2014). Efficacy of certolizumab pegol on signs and symptoms of axial spondyloarthritis including ankylosing spondylitis: 24-week results of a double-blind randomised placebo-controlled Phase 3 study. Ann Rheum Dis..

[18] Song IH, Weiss A, Hermann KG, Haibel H, Althoff CE, Poddubnyy D, Listing J, Lange E, Freundlich B, Rudwaleit M, Sieper J (2013). Similar response rates in patients with ankylosing spondylitis and non-radiographic axial spondyloarthritis after 1 year of treatment with etanercept: results from the ESTHER trial. Ann Rheum Dis..

[19] Pham T, Fautrel B, Dernis E, Goupille P, Guillemin F, Le Loët X, Ravaud P, Claudepierre P, Miceli-Richard C, De Bandt M, Breban M (2007). Recommendations of the French Society for Rheumatology regarding TNFα antagonist therapy in patients with ankylosing spondylitis or psoriatic arthritis: 2007 update. Joint Bone Spine..

[20] Dougados M, d’Agostino MA, Benessiano J, Berenbaum F, Breban M, Claudepierre P, Combe B, Dargent-Molina P, Daures JP, Fautrel B, Feydy A, Goupille P, Leblanc V, Logeart I, Pham T, Richette P, Roux C, Rudwaleit M, Saraux A, Treluyer JM, van der Heijde D, Wendling D (2011). The DESIR cohort: a 10-year follow-up of early inflammatory back pain in France: study design and baseline characteristics of the 708 recruited patients. Joint Bone Spine..

[21] Costantino F, Aegerter P, Dougados M, Breban M, D’Agostino MA (2016). Two phenotypes are identified by cluster analysis in early inflammatory back pain suggestive of Spondyloarthritis: results from the DESIR Cohort. Arthritis Rheumatol..

[22] Desthieux C, Molto A, Granger B, Saraux A, Fautrel B, Gossec L (2016). Patient-physician discordance in global assessment in early spondyloarthritis and its change over time: the DESIR cohort. Ann Rheum Dis..

[23] Molto A, Tezenas du Montcel S, Wendling D, Dougados M, Vanier A, Gossec L (2016). Disease activity trajectories in early axial spondyloarthritis: results from the DESIR cohort. Ann Rheum Dis..

[24] Calin A, Porta J, Fries JF, Schurman DJ (1977). Clinical history as a screening test for ankylosing spondylitis. JAMA..

[25] Rudwaleit M, Metter A, Listing J, Sieper J, Braun J (2006). Inflammatory back pain in ankylosing spondylitis: a reassessment of the clinical history for application as classification and diagnostic criteria. Arthritis Rheum..

[26] Harvard S, Guh D, Bansback N, Richette P, Dougados M, Anis A, Fautrel B (2016). Costs of early spondyloarthritis: estimates from the first 3 years of the DESIR cohort. RMD Open..

[27] National Institute of Statistics and Economic Studies. Publications et statistiques pour la France ou les régions. http://www.insee.fr/fr/themes/theme.asp?theme=4. Accessed Mar 3 2015.

[28] Maksymowych WP, Gladman D, Rahman P, Boonen A, Bykerk V, Choquette D, Dimond S, Fortin P, Karsh J, Klinkhoff AV, Mosher D, Mulholland K, Olszynski WP, Russell AS, Savage L, Shanner L, Shojania K, Starr M, Thomson G, Zummer M, Inman R (2007). Canadian Rheumatology Association/Spondyloarthritis Research Consortium of Canada. The Canadian Rheumatology Association/Spondyloarthritis Research Consortium of Canada treatment recommendations for the management of spondyloarthritis: a national multidisciplinary stakeholder project. J Rheumatol..

[29] Kiltz U, Sieper J, Kellner H, Krause D, Rudwaleit M, Chenot JF, Stallmach A, Jaresch S, Braun J (2014). German Society for Rheumatology. German Society for Rheumatology S3 guidelines on axial spondyloarthritis including Bechterew’s disease and early forms: 8.4 Pharmaceutical therapy, 8.5 Evaluation of therapy success of pharmaceutical measures. Z Rheumatol..

[30] Mok CC (2005). Consensus statements on the indications and monitoring of anti-tumor necrosis factor (TNF) therapy for rheumatic diseases in Hong Kong. Hong Kong Bull Rheum Dis.

[31] Keat A, Barkham N, Bhalla A, Gaffney K, Marzo-Ortega H, Paul S, Rogers F, Somerville M, Sturrock R, Wordsworth P, BSR Standards, Guidelines and Audit Working group (2005). BSR guidelines for prescribing TNF-alpha blockers in adults with ankylosing spondylitis. Report of a working party of the British Society for Rheumatology. Rheumatology (Oxford).

[32] van der Heijde D, Sieper J, Maksymowych WP, Dougados M, Burgos-Vargas R, Landewé R, Rudwaleit M, Braun J, Assessment of SpondyloArthritis international Society (2010). Update of the international ASAS recommendations for the use of anti-TNF agents in patients with axial spondyloarthritis. Ann Rheum Dis..

[33] Brazier J, Roberts J, Deverill M (2002). The estimation of a preference-based measure of health from the SF-36. J Health Econ..

[34] Whitehead SJ, Ali S (2010). Health outcomes in economic evaluation: the QALY and utilities. Br Med Bull..

[35] Dias S, Sutton AJ, Welton NJ, Ades AE (2013). Evidence synthesis for decision making 6: embedding evidence synthesis in probabilistic cost-effectiveness analysis. Med Decis Mak.

[36] Costantino F, Talpin A, Said-Nahal R, Goldberg M, Henny J, Chiocchia G, Garchon HJ, Zins M, Breban M (2015). Prevalence of spondyloarthritis in reference to HLA-B27 in the French population: results of the GAZEL cohort. Ann Rheum Dis..

[37] Gaujoux-Viala C, Fautrel B (2012). Cost effectiveness of therapeutic interventions in ankylosing spondylitis: a critical and systematic review. Pharmacoeconomics..

[38] Arends S, Brouwer E, van der Veer E, Groen H, Leijsma MK, Houtman PM, Th A, Jansen TL, Kallenberg CG, Spoorenberg A (2011). Baseline predictors of response and discontinuation of tumor necrosis factor-alpha blocking therapy in ankylosing spondylitis: a prospective longitudinal observational cohort study. Arthritis Res Ther..

[39] Lord PA, Farragher TM, Lunt M, Watson KD, Symmons DP, Hyrich KL, BSR Biologics Register (2010). Predictors of response to anti-TNF therapy in ankylosing spondylitis: results from the British society for rheumatology biologics register. Rheumatology (Oxford).

[40] Rudwaleit M, Listing J, Brandt J, Braun J, Sieper J (2004). Prediction of a major clinical response (BASDAI 50) to tumour necrosis factor alpha blockers in ankylosing spondylitis. Ann Rheum Dis..

[41] Vastesaeger N, van der Heijde D, Inman RD, Wang Y, Deodhar A, Hsu B, Rahman MU, Dijkmans B, Geusens P, Vander Cruyssen B, Collantes E, Sieper J, Braun J (2011). Predicting the outcome of ankylosing spondylitis therapy. Ann Rheum Dis..

[42] Glintborg B, Sorensen IJ, Ostergaard M, Dreyer L, Mohamoud AA, Krogh NS, Hendricks O, Andersen LS, Raun JL, Kowalski MR, Danielsen L, Pelck R, Nordin H, Pedersen JK, Kraus DG, Christensen SR, Hansen IM, Esbesen J, Schlemmer A, Loft AG, Al Chaer N, Salomonsen L, Hetland ML (2017). Ankylosing Spondylitis versus nonradiographic axial Spondyloarthritis: comparison of tumor necrosis factor inhibitor effectiveness and effect of HLA-B27 status. An observational cohort study from the Nationwide DANBIO Registry. J Rheumatol..

[43] Sieper J, Landewe R, Rudwaleit M, van der Heijde D, Dougados M, Mease PJ, Braun J, Deodhar A, Kivitz A, Walsh J, Hoepken B, Nurminen T, Maksymowych WP (2015). Effect of certolizumab pegol over ninety-six weeks in patients with axial spondyloarthritis: results from a phase III randomized trial. Arthritis Rheumatol..

[44] Brandt J, Listing J, Sieper J, Rudwaleit M, van der Heijde D, Braun J (2004). Development and preselection of criteria for short term improvement after anti-TNF alpha treatment in ankylosing spondylitis. Ann Rheum Dis..

[45] Lukas C, Landewe R, Sieper J, Dougados M, Davis J, Braun J, van der Linden S, Heijde D (2009). Assessment of SpondyloArthritis international Society. Development of an ASAS-endorsed disease activity score (ASDAS) in patients with ankylosing spondylitis. Ann Rheum Dis..

[46] van der Heijde D, Joshi A, Pangan AL, Chen N, Betts K, Mittal M, Bao Y (2016). ASAS40 and ASDAS clinical responses in the ABILITY-1 clinical trial translate to meaningful improvements in physical function, health-related quality of life and work productivity in patients with non-radiographic axial spondyloarthritis. Rheumatology (Oxford)..

[47] Walters SJ, Brazier JE (2003). What is the relationship between the minimally important difference and health state utility values? The case of the SF-6D. Health Qual Life Outcomes..

[48] Walters SJ, Brazier JE (2005). Comparison of the minimally important difference for two health state utility measures: EQ-5D and SF-6D. Qual Life Res..

[49] Sieper J, Lenaerts J, Wollenhaupt J, Rudwaleit M, Mazurov VI, Myasoutova L, Park S, Song Y, Yao R, Chitkara D, Vastesaeger N, All INFAST Investigators (2014). Efficacy and safety of infliximab plus naproxen versus naproxen alone in patients with early, active axial spondyloarthritis: results from the double-blind, placebo-controlled INFAST study, Part 1. Ann Rheum Dis..

[50] Drummond M (2016). Clinical guidelines: a NICE way to introduce cost-effectiveness considerations?. Value Health..

[51] Antioch KM, Drummond MF, Niessen LW, Vondeling H (2017). International lessons in new methods for grading and integrating cost effectiveness evidence into clinical practice guidelines. Cost Eff Resour Alloc..

