# Supplementary material for: Access criteria for anti-TNF agents in spondyloarthritis: influence on comparative 1-year cost-effectiveness estimates
Source: Cost Eff Resour Alloc. 2017 Sep 7;15:20. doi: 10.1186/s12962-017-0081-8 (PMC5590198; doi:10.1186/s12962-017-0081-8)
Supplement: Supplementary file 1 — Additional file 1: Table S1. Characteristics of anti-TNF users not satisfying selected criteria sets. [file 12962_2017_81_MOESM1_ESM.docx]

**Additional Table S1. Characteristics of anti-TNF users not satisfying selected criteria sets**

|  | **Not Satisfying Any (n=94)** | **Not Satisfying Canada (n=118)** | **Not Satisfying France (n=107)** | **Not Satisfying UK (n=177)** | **Not Satisfying Germany (n=120)** | **Not Satisfying Hong Kong (n=188)** |
| --- | --- | --- | --- | --- | --- | --- |
| ***At baseline*** |  |  |  |  |  |  |
| Age | 34.14 ± 8.48 | 34.35 ± 8.76 | 34.41 ± 8.64 | 34.3 ± 8.78 | 34.62 ± 8.72 | 34.21 ± 8.89 |
| Male | 35 (37.2%) | 41 (34.7%) | 38 (35.5%) | 69 (39%) | 45 (37.5%) | 77 (41%) |
| Post-secondary education | 51 (54.3%) | 60 (50.8%) | 55 (51.4%) | 95 (53.7%) | 64 (53.3%) | 101 (53.7%) |
| Married | 59 (62.8%) | 75 (63.6%) | 69 (64.5%) | 113 (63.8%) | 75 (62.5%) | 118 (62.8%) |
| Smoking | 33 (35.1%) | 42 (35.6%) | 39 (36.4%) | 69 (39%) | 41 (34.2%) | 72 (38.3%) |
| Disease Duration | 1.47 ± 0.88 | 1.46 ± 0.88 | 1.45 ± 0.89 | 1.52 ± 0.88 | 1.47 ± 0.85 | 1.53 ± 0.87 |
| Peripheral Arthritis | 66 (70.2%) | 84 (71.2%) | 76 (71%) | 127 (71.8%) | 83 (69.2%) | 133 (70.7%) |
| HLA: Positive | 45 (47.9%) | 60 (50.8%) | 55 (51.4%) | 95 (53.7%) | 51 (42.5%) | 104 (55.3%) |
| Radiographic sacroiliitis | 12 (12.8%) | 20 (16.9%) | 16 (15%) | 39 (22%) | 20 (16.7%) | 47 (25%) |
| Sacroiilitis or spine inflammation on MRI | 14 (14.9%) | 17 (14.4%) | 18 (16.8%) | 59 (33.3%) | 14 (11.7%) | 65 (34.6%) |
| BASDAI ≥4 | 69 (73.4%) | 92 (78%) | 80 (74.8%) | 144 (81.4%) | 94 (78.3%) | 152 (80.9%) |
| CRP >10 mg/L | 22 (23.4%) | 25 (21.2%) | 25 (23.4%) | 42 (23.7%) | 26 (21.7%) | 49 (26.1%) |
| Physician's Global Assessment ≥4 | 72 (76.6%) | 90 (76.3%) | 79 (73.8%) | 142 (80.2%) | 94 (78.3%) | 150 (79.8%) |
| Inflammatory back pain ≥4 | 81 (86.2%) | 102 (86.4%) | 94 (87.9%) | 157 (88.7%) | 103 (85.8%) | 168 (89.4%) |
| Morning stiffness ≥45 mins | 51 (54.3%) | 70 (59.3%) | 60 (56.1%) | 99 (55.9%) | 68 (56.7%) | 100 (53.2%) |
| Patient Global Assessment ≥4 | 81 (86.2%) | 104 (88.1%) | 93 (86.9%) | 156 (88.1%) | 106 (88.3%) | 165 (87.8%) |
| ***At time of treatment initiation*** |  |  |  |  |  |  |
| BASDAI | 42.26 ± 22.35 | 44.44 ± 21.9 | 42.84 ± 22.3 | 41.87 ± 22.44 | 44.2 ± 21.92 | 40.92 ± 22.56 |
| BASFI | 32.16 ± 23.2 | 34.52 ± 23.24 | 33.48 ± 23.26 | 32.28 ± 22.69 | 34.19 ± 22.52 | 32.07 ± 22.58 |
| SF-36 | 0.62 ± 0.12 | 0.61 ± 0.12 | 0.62 ± 0.12 | 0.62 ± 0.12 | 0.61 ± 0.12 | 0.63 ± 0.12 |
| Physician's Global Assessment | 3.85 ± 2.57 | 3.98 ± 2.59 | 3.81 ± 2.61 | 3.76 ± 2.58 | 4.02 ± 2.52 | 3.72 ± 2.61 |
| CRP | 6.53 ± 17.51 | 6.03 ± 15.71 | 6.44 ± 16.49 | 5.63 ± 14.01 | 5.96 ± 15.78 | 6 ± 14.33 |
| *Statistics presented are: Mean ± SD, or N (%) | | | | | | |
