# Supplementary material for: Access criteria for anti-TNF agents in spondyloarthritis: influence on comparative 1-year cost-effectiveness estimates
Source: Cost Eff Resour Alloc. 2017 Sep 7;15:20. doi: 10.1186/s12962-017-0081-8 (PMC5590198; doi:10.1186/s12962-017-0081-8)
Supplement: Supplementary file 2 — Additional file 2: Table S2. Total time on anti-TNF therapy among non-responders (from initiation until end of follow-up). [file 12962_2017_81_MOESM2_ESM.docx]

**Additional Table S2. Total time on anti-TNF therapy among non-responders (from initiation until end of follow-up)**

| **Interval** | **Canada (N=32)** | **France (N=38)** | **UK (N=23)** | **Germany (N=32)** | **Hong Kong (N=19)** |
| --- | --- | --- | --- | --- | --- |
| < 12weeks |  | 1 ( 2.6%) |  |  |  |
| 12-24 weeks | 0 ( 0.0%) | 0 ( 0.0%) | 0 ( 0.0%) | 2 ( 6.3%) | 0 ( 0.0%) |
| 24 weeks to 1 year | 1 ( 3.1%) | 1 ( 2.6%) | 1 ( 4.3%) | 1 ( 3.1%) | 1 ( 5.3%) |
| 1-2 years | 11 (34.4%) | 13 (34.2%) | 7 (30.4%) | 10 (31.3%) | 7 (36.8%) |
| 2-3 years | 13 (40.6%) | 15 (39.5%) | 9 (39.1%) | 13 (40.6%) | 5 (26.3%) |
| 3-4 years | 7 (21.9%) | 8 (21.1%) | 6 (26.1%) | 6 (18.8%) | 6 (31.6%) |
