# Supplementary material for: Access criteria for anti-TNF agents in spondyloarthritis: influence on comparative 1-year cost-effectiveness estimates
Source: Cost Eff Resour Alloc. 2017 Sep 7;15:20. doi: 10.1186/s12962-017-0081-8 (PMC5590198; doi:10.1186/s12962-017-0081-8)
Supplement: Supplementary file 3 — Additional file 3: Table S3. Comparative estimates of costs, QALYs, and ICERs in sensitivity analyses. [file 12962_2017_81_MOESM3_ESM.docx]

**Additional Table S3. Comparative estimates of costs, QALYs, and ICERs in sensitivity analyses**

| **Basecase population, excluding indirect costs** | | | | | | |
| --- | --- | --- | --- | --- | --- | --- |
|  |  | **Anti-TNF User** | **Anti-TNF Non-User** | **Incremental Cost** | **Incremental QALY** | **ICER** |
| Canada | Cost | 14,704 | 1,284 | 13,420 | 0.017 | 782,141 |
| Canada | Qaly | 0.626 | 0.609 |  |  |  |
| France | Cost | 14,777 | 1,286 | 13,490 | 0.013 | 1,064,838 |
| France | Qaly | 0.620 | 0.607 |  |  |  |
| UK | Cost | 14,920 | 979 | 13,941 | 0.021 | 659,561 |
| UK | Qaly | 0.627 | 0.606 |  |  |  |
| Germany | Cost | 14,652 | 1,378 | 13,273 | 0.025 | 527,169 |
| Germany | Qaly | 0.642 | 0.617 |  |  |  |
| HK | Cost | 14,177 | 1,013 | 13,164 | 0.033 | 399,813 |
| HK | Qaly | 0.619 | 0.586 |  |  |  |
| **Including anti-TNF users who received therapy prior to criteria satisfaction (rule 1 lifted)** | | | | | | |
| Canada | Cost | 16,319 | 1,837 | 14,482 | -0.023 | Dominated |
| Canada | Qaly | 0.609 | 0.631 |  |  |  |
| France | Cost | 15,952 | 2,073 | 13,879 | -0.023 | Dominated |
| France | Qaly | 0.604 | 0.627 |  |  |  |
| UK | Cost | 16,783 | 1,556 | 15,227 | -0.014 | Dominated |
| UK | Qaly | 0.614 | 0.628 |  |  |  |
| Germany | Cost | 16,476 | 1,850 | 14,626 | -0.016 | Dominated |
| Germany | Qaly | 0.625 | 0.641 |  |  |  |
| HK | Cost | 16,200 | 1,498 | 14,702 | -0.016 | Dominated |
| HK | Qaly | 0.597 | 0.614 |  |  |  |
| **Including anti-TNF users who received therapy 6-12 months after criteria satisfaction (rule 2 lifted)** | | | | | | |
| Canada | Cost | 16,254 | 2,071 | 14,182 | -0.027 | Dominated |
| Canada | Qaly | 0.608 | 0.635 |  |  |  |
| France | Cost | 16,091 | 2,256 | 13,835 | -0.028 | Dominated |
| France | Qaly | 0.603 | 0.631 |  |  |  |
| UK | Cost | 16,166 | 1,784 | 14,382 | -0.021 | Dominated |
| UK | Qaly | 0.609 | 0.630 |  |  |  |
| Germany | Cost | 15,726 | 2,060 | 13,666 | -0.023 | Dominated |
| Germany | Qaly | 0.617 | 0.639 |  |  |  |
| HK | Cost | 15,939 | 1,708 | 14,231 | -0.020 | Dominated |
| HK | Qaly | 0.596 | 0.616 |  |  |  |
| **Including anti-TNF users who received therapy prior to or 6-12 months after criteria satisfaction (rule 1 and 2 lifted)** | | | | | | |
| Canada | Cost | 16,216 | 2,065 | 14,152 | -0.030 | Dominated |
| Canada | Qaly | 0.605 | 0.635 |  |  |  |
| France | Cost | 15,940 | 2,227 | 13,713 | -0.028 | Dominated |
| France | Qaly | 0.602 | 0.630 |  |  |  |
| UK | Cost | 16,155 | 1,796 | 14,360 | -0.021 | Dominated |
| UK | Qaly | 0.608 | 0.629 |  |  |  |
| Germany | Cost | 15,933 | 1,994 | 13,938 | -0.024 | Dominated |
| Germany | Qaly | 0.616 | 0.640 |  |  |  |
| HK | Cost | 15,767 | 1,747 | 14,020 | -0.020 | Dominated |
| HK | Qaly | 0.592 | 0.612 |  |  |  |
